# Urinary Tract Infections Detection with Molecular Biomarkers

**DOI:** 10.3390/biom14121540

**Published:** 2024-11-30

**Authors:** Jiayi Sun, Kai Cheng, Yanyun Xie

**Affiliations:** 1 Xiangya Hospital, Central South University, Changsha 410008, China; sunjiayi@csu.edu.cn; 2 Department of Urology, Xiangya Hospital, Central South University, Changsha 410008, China; kai_cheng@csu.edu.cn; 3 Department of Nephrology, Xiangya Hospital, Central South University, Changsha 410008, China

**Keywords:** urinary tract infections, biomarkers, pathogens, diagnosis

## Abstract

Urinary tract infection (UTI) is the most prevalent kind of pathogenic bacteria infection, and the midstream urine culture is regarded as the gold standard in UTI diagnosis. Recently, even with modern media and techniques such as polymerase chain reaction (PCR), urinary cultures still create a considerable workload for hospital laboratories. Other UTI-detecting methods, such as flow cytometry and lateral flow immunoassay, suffer from various drawbacks like long time consumption and low sensitivity. Therefore, looking for reliable biomarkers in UTI is urgently needed. In this review, the current definitions of UTI can be basically divided into two main categories: uncomplicated UTI and complicated UTI. In light of anatomical sites, it can be classified as either lower UTI or upper UTI. We take the classification of UTI as a clue and review the reported extensive literature to classify the existing studied markers into the following three categories: Biomarkers used clinically; Promising biomarkers; and Controversial biomarkers. Particularly, the nucleic acid-associated, metabolomic, and lipidomic biomarkers are highlighted. At the end, we discuss the challenges and prospects of biomarkers in UTI, hoping to further inspire the diagnosis of UTI.

## 1. Introduction

Urinary tract infection (UTI) has already become a substantial public-health issue in all age groups, contributing to more than 6.8 million office visits and 1.3 million emergency department visits every year in the United States and ultimately leading to almost 100,000 hospitalizations [1]. Microbiologically, UTI is the specific inflammatory response of the uroepithelium to infectious agents. However, the bacteriology of UTI is truly predictable, in which the Gram-negative, facultatively anaerobic, and uropathogenic *Escherichia coli* (UPEC) account for the most infections. Though urine culture has been a common diagnostic method for UTI, it is still a non-negligible issue that urinary symptoms and bacteriuria are often independent of each other. There are some studies reported that approximately 20% of women appearing to have typical UTI symptoms show negative urine cultures, while different kinds of bacteria are invariably examined in asymptomatic or healthy individuals [2]. Accordingly, the combination of urinary symptoms and urine culture for the diagnosis has vital significance.

**Figure 1:**
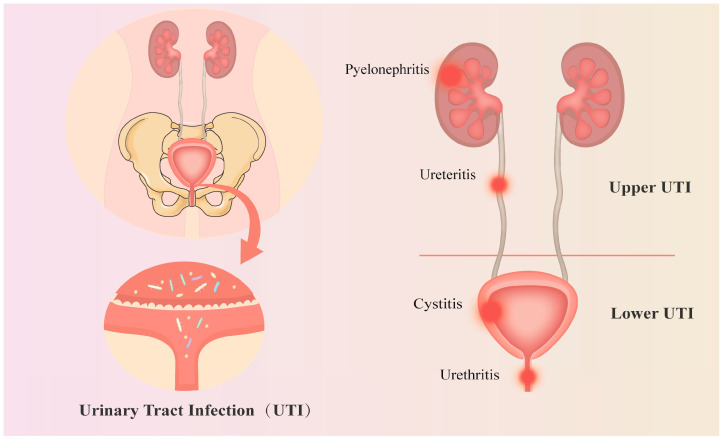
The pathogenesis and anatomical classification of UTI. UTI is mainly caused by the infection of bacteria or other microbes. Taking the bladder as a dividing line, UTI includes the upper UTI (pyelonephritis and ureteritis) and the lower UTI (cystitis, urethritis, and prostatitis).

The current definitions of UTI can be divided into two main categories: uncomplicated UTI and complicated UTI [3]. Uncomplicated UTI refers to abacterial infections of the bladder and correlated structures. Patients with uncomplicated UTI seldom present structural abnormalities or comorbidities; for instance, typical diseases include diabetes, immunocompromised states, and pregnancy [4]. Whereas, complicated UTI is often combined with the following conditions: urinary tract stones, obstruction, deformity, polycystic kidney, neurogenic bladder, renal function injury, or foreign bodies [5]. According to the anatomical sites, UTI can be classified as either lower UTI or upper UTI (Figure 1). Upper UTI includes the complicated UTI like pyelonephritis. While the lower UTI is often shown through cystitis, which is the most common form of UTI [6].

UTI influences almost 150 million people worldwide every year, and females are more likely to suffer from UTI because of their short and wide urethra, whose opening is below the labia, close to the external opening of the vagina and anus [7]. Indeed, it was estimated more than 50% of all women have ever experienced UTI throughout their lifetime, and approximately 50% of them may obtain recurrent infections in the following 6 months [8,9]. In addition to adults, the prevalence of UTI has also reached 3.3% and 6.5%, respectively, in male and female babies [10].

With high prevalence, UTI plays a crucial role in hospital-acquired infection and subsequent morbidity and inevitably accounts for lots of outpatients and emergency department consultations, tremendously highlighting the public health burden [9]. Nevertheless, antimicrobial resistance is becoming riskier than these factors, which is now one of the major threats to patient safety worldwide, bringing about more serious infections, longer hospital stays, and raised mortality. Therefore, making a more accurate and rapid diagnosis of UTI has vital significance, able to effectively reduce the abuse of antibiotics and reduce antibiotic resistance. Urinary culturing is recognized as the gold standard for UTI diagnosis, possessing various disadvantages, including a low positive determination rate and being time-consuming, which is unable to meet clinical needs. Accordingly, finding new biomarkers of UTI applied to diagnosis is of great value.

Here, we take the classification of UTI as a clue for reviewing the reported extensive literature to classify the existing studied markers into the following three categories: biomarkers used clinically, promising biomarkers, and controversial biomarkers. And the promising biomarkers are divided into four categories, including protein and peptide markers, nucleic acid-associated markers, glycosylated markers, and metabolomic and lipidomic markers. Finally, we discuss the challenges and prospects of existing biomarkers, which we hope will further enlighten the diagnosis of UTI.

## 2. Biomarkers Used Clinically

Referring to the current clinical diagnostic criteria for UTI, we describe the markers that have been used in this section and also introduce the relatively new clinical marker NGAL (Table 1).

### 2.1. Conventional Biomarker

#### 2.1.1. PCT and CRP

Procalcitonin (PCT) is a pro-peptide of calcitonin and normally presents in plasma with a concentration of about 6 mg/L but rises in inflammation [23]. Through analyzing the levels of serum PCT in 293 UTI patients, Levine et al. revealed that PCT < 0.25 ng/mL could exclude UTI, and screening biomarkers may enable effective treatment of UTI and minimize the abuse of antibiotics [24]. Another study identified that serum PCT and C-reactive protein (CRP) could be utilized as biomarkers for differentiating upper UTI and lower UTI in children, while the sensitivity (90.47%) and specificity (88.0%) of PCT were better than that of CRP (sensitivity was 85.7%, specificity was 48%), revealing that PCT is a more suitable biomarker for differential diagnosis in children [17]. However, Kuil et al. have shown that neither CRP nor PCT is appropriate for distinguishing between UTI and asymptomatic bacteriuria (ASB) in nursing home residents, for the sensitivities of CRP and PCT were 52.3% and 37.0%, respectively [25]. Furthermore, PCT plays a limited role for diagnosing UTI in hospitalized older adults [26]. A recent study found that combined detection of serum CRP and PCT is more beneficial for diagnosing bacterial UTI. Both CRP and PCT demonstrate high sensitivity and negative predictive value (NPV) in diagnosing bacterial UTI caused by Candida infection [27]. Recent findings have demonstrated PCT can safely exclude bacteremia in clinically relevant numbers in emergency department patients with suspected UTI [28]. Of note, Shaikh et al. reported that it is possible to use CRP levels (<20 mg/L) to exclude pyelonephritis [18]. Another meta-analysis found that PCT has a role in predicting pyelonephritis, with a sensitivity of 74% and a specificity of 76% at a cut-off of 71.67 ng/mL [19]. Meanwhile, a meta-analysis of 18 studies assessing the diagnostic value of PCT in acute pyelonephritis (APN) included 831 patients with APN and 651 with lower UTI. The findings indicated that a serum PCT level of ≥0.5 ng/mL had a pooled sensitivity of 0.86 and a pooled specificity of 0.76 [29].

#### 2.1.2. IL-1β

IL-1β, first discovered in the free form of different biological fluids, is regarded as a paramount immune regulatory cytokine and plays a role in distinguishing between the lower UTI and APN. In 2005, Gurgoze and colleagues compared the levels of serum IL-1β in children with lower UTI or pyelonephritis. It is demonstrated that when the cutoff of 6.9 pg/mL was applied, the sensitivity and specificity of IL-1β, respectively, in diagnostics of upper UTI reached 97% and 59% [11]. For febrile children, urine IL-1β was also proved to be an effective biomarker for early detecting APN. Additionally, initial urinary IL-1β levels were significantly higher in children with APN than in controls with low UTI and non-renal fever. Urinary IL-1β was positively correlated with fever, CRP, leukocytes, neutrophils, and leukocyturia in children with APN [12]. A recent study has demonstrated that IL-1β will be released by renal fibroblasts during UPEC infection, which may be one of the mechanisms for the increased concentration of IL-1β during UTI [30].

#### 2.1.3. IL-6

IL-6 is released in inflammatory states by WBCs and urothelial cells, with a wide range of functions, presenting an important part in the anti-infection immune responses [31]. In line, IL-6 drives specific transcriptional programs of the antimicrobial gene in infected urothelial cells, effectively preventing epithelial invasion and ascending infection [32]. Due to its pivotal effect, IL-6 can also be investigated as a biomarker for predicting UTI. In one study, IL-6 was the only biomarker that was elevated in patients with ASB when UTI occurred, and the sensitivity and specificity of IL-6 (critical value of 25 pg/mL) to differentiate between ASB and UTI were 57% and 80%, respectively, manifesting that urinary IL-6 is promising in the detection between ASB and symptomatic UTI in the elderly [15]. In detail, the urine IL-6 concentration was >20 pg/mL and the renal volume was elevated, indicating APN. However, the opposite conclusion was gained by Rushood and his team, reporting that neither urine nor serum IL-6 could distinguish APN and lower UTI in children [33]. Moreover, the latest study stated that IL-6 might mediate a delirium-like phenotype in the mouse model of UTI, providing a preclinical rationale for clinical studies of IL-6 inhibitors for the treatment of delirium-induced by UTI [34]. Both Darogha et al. and Hosseini et al. recently verified that elevated IL-6 levels were predictive for the diagnosis of UTI in both children and adults [13,14].

#### 2.1.4. IL-8

IL-8 is a cytokine secreted by macrophages and epithelial cells, possessing the chemotactic effect on neutrophils to realize its regulation of inflammatory response. To date, IL-8 is commonly regarded as a potential diagnostic marker in UTI [35]. It was first reported that urinary IL-8 was substantially increased in UTI patients versus healthy people [36]. Urine IL-8 (cutoff 200 pg/mL) is a sensitive biomarker of UTI diagnosis, with the sensitivity and specificity being 93% and 90%, respectively [16]. Kajbaf et al. also demonstrated the diagnostic value of urinary IL-8 assay in differentiating pyelonephritis from cystitis in children, and the results presented that IL-8/Cr assay also possessed sensitivity and specificity for diagnosing pyelonephritis, which referred to 66% and 72%, respectively. In addition, the diagnostic utility of IL-8 urine levels in detecting febrile UTI in children is preferable to serum IL-8 levels [14]. A recent study found that urine IL-8 offered no additional diagnostic value beyond routine tests, except for a slight enhancement in accuracy for patients with leukopenia [37]. Meanwhile, in postmenopausal women with recurrent UTI, IL-8 may act as a biomarker to enhance diagnosis [38]. With the relatively low specificity, IL-8 levels increase in every kind of congenital urinary abnormality in addition to prenatal pyelitis. Therefore, IL-8 is not suitable for the diagnosis of UTI when anatomical disorders are present [39].

### 2.2. Newly Used Biomarkers—NGAL

Neutrophil gelatinase-associated lipocalin (NGAL) is commonly found in neutrophils and many other body tissues, such as alpha-intercalated cells in renal collecting ducts. NGAL chelates iron from gram-negative bacteria to play the bacteriostatic effect [40]. The study reviewed the correlation between NGAL levels and UTI in 260 children with UTI aged 3 to 24 months, revealing that the urine NGAL used for UTI diagnosis has a sensitivity and specificity of 97.1% and 95.6%, respectively. Studies have shown urine NGAL remarkably decreased in those patients with recurrent UTI, and children with reduced urinary NGAL excretion are more commonly with recurrent febrile UTI [20]. Although some scholars have suggested that NGAL is predictive of recurrent UTI, initially occurring renal scarring should be ruled out [21]. Moreover, Jung et al. demonstrated that the level of NGAL in the urine of children with UTI was much higher than that of non-UTI, but there was no difference in the levels of β2-microglobulin between the two groups [41]. Recent research represented that the diagnostic sensitivity and specificity of urinary NGAL for UTI are at high levels in young febrile children, with values of 90.3% and 93.7%, respectively [22]. Hence, NGAL is a biomarker of essential clinical significance for the diagnosis of UTI both in adults and infants.

## 3. Promising Biomarkers

**Figure 2:**
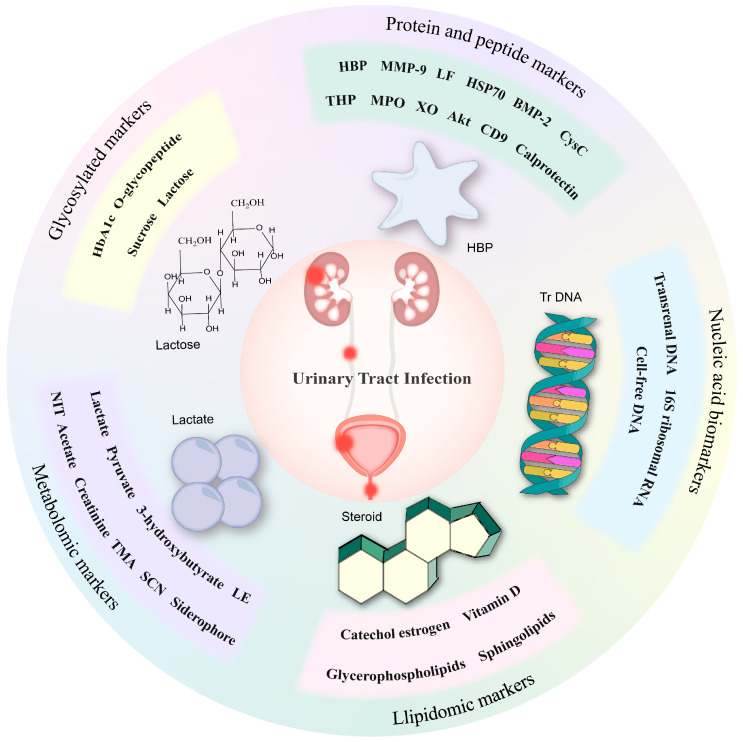
Category of promising biomarkers for UTI. The promising biomarkers are divided based on different biochemical compositions, including protein and peptide markers, nucleic acid biomarkers, glycosylated markers, metabolomic markers, and lipidomic markers.

According to current studies on markers of UTI, we screened some biomarkers that are considered to have diagnostic potential and elaborated on them in terms of different biochemical compositions (Figure 2).

### 3.1. Protein and Peptide Markers

#### 3.1.1. HBP

Heparin-binding protein (HBP) originates from the secretory granules and azurophilic granules in neutrophils and has been investigated as the biomarker of many potential bacterial infections. It can be proved that the significant increase in HBP levels in urine reflects the severity of UTI. Furthermore, urinary HBP is considered a more reliable indicator of UTI than urinary leukocytes or urinary IL-6 levels [42]. There was an experiment that demonstrated urinary HBP might become the best biomarker to distinguish between lower UTI and pyelonephritis versus urinary IL-6, leukocytes, and nitrite [43].

#### 3.1.2. MMP-9

Matrix metalloproteinases-9 (MMP-9) acts specifically in the turnover and degradation of extracellular matrix as a metalloprotease enzyme and is responsible for regulating inflammation and immunity [44]. Hatipoglu et al. detected mean urinary MMP-9/NGAL/Cr concentrations in the UTI group to be markedly higher than in the control group among children. They demonstrated that the sensitivity and specificity of urine MMP-9/NGAL/Cr concentrations to predict UTI were 98% and 97%, respectively, at the cut-off value of 0.08 ng/mg, indicating the urinary MMP-9/NGAL complex could have a better diagnostic performance than NGAL alone. The urinary MMP-9/NGAL complex may be useful for distinguishing ASB from UTI for its high sensitivity and specificity [45]. Moreover, there are also studies that have demonstrated the clinical value of urinary MMP-9 and tissue inhibitor of metalloproteinase 1 (TIMP1) levels as novel biomarkers in identifying the risk of vesicoureteral reflux (VUR) and renal scar formation in children. MMP-9 seemingly is unable to distinguish renal scars from VUR, but the simultaneous increase of these two biomarker levels may indicate sustained renal damage caused by VUR [46]. In APN children, elevated levels of urinary MMP-9 and TIMP1 may help identify the risk of renal scar formation with 75% sensitivity and 60.5% specificity [47].

#### 3.1.3. LF

Lactoferrin (LF) released from polymorphonuclear leukocytes, urinary LF is considered a prospective biomarker for diagnosing UTI. A study found taking 200 ng/mL as the cut-off value, urinary LF distinguished ASB from UTI with 93% sensitivity and 89% specificity [48]. In a current study, bovine lactoferrin could be used as a safe and effective treatment for recurrent cystitis [49]. The value of LF in diagnosing and treating UTI should be the subject of further research.

#### 3.1.4. HSP70

Heat shock protein-70 (HSP70) is upregulated when the cells are exposed to stress situations such as infectious agents to avoid alterations in the structure of proteins. Yilmaz et al. observed that the urine HSP70/Cr ratio preceding therapy was considerably higher in the children with UTI than in the control group. Due to its excellent specificity and sensitivity, HSP70 may assist in distinguishing UTI from ASB along with additional infections [50]. Additionally, the urinary HSP70 showed 90% sensitivity and 82% specificity for diagnosing among the 265 children with suspected UTI, indicating better individual diagnostic accuracy than nitrite and leukocyte esterase positivity [51].

#### 3.1.5. BMP-2, CysC, THP

Bone morphogenic protein-2 (BMP-2) significantly rises in urinary stone formation and infection. Cystatin C (CysC), holding predictive value in diagnosing APN in children, is an endogenous biomarker for kidney injury. A study found the combined application of serum BMP-2 and CysC has better sensitivity and accuracy for the early diagnosis of kidney infection. With a cut-off of 44 pg/mL, the specificity of serum BMP-2 was 80%, and the sensitivity was 92%. With a cut-off of 525 ng/mL, the specificity of serum CysC was 91%, and the sensitivity was 85%. Besides, Tamm-Horsfall protein (THP) is the protein most commonly found in the urine of healthy people, and it also possesses a high diagnostic value for UTI, with 75% specificity and 94% sensitivity, taking 305 ng/mL as a cut-off value [52].

#### 3.1.6. MPO

Myeloperoxidase (MPO) is a candidate biomarker of inflammation. Some studies revealed that the activities of MPO are elevated in a series of kidney diseases. Likewise, Ciragi et al. found it markedly went up in the urine of UTI patients, with 87% sensitivity and 100% specificity [53]. Furthermore, the urinary MPO/creatinine ratio may be a prospective marker for observing the treatment response in UTI [54]. According to the latest study, MPO as a marker of urinary neutrophil extracellular traps (NETs) had markedly higher concentrations in children with UTI, supporting the hypothesis of a correlation between UTI in children and NETs [55].

#### 3.1.7. XO

Human xanthine oxidase (XO) is mainly found in the liver. The activities of urinary XO have been proven with UTI raised, and only when the urine contained bacteria > 105/mL, the activities of XO increased significantly. The urinary XO to diagnose UTI can attain 100% in both sensitivity and specificity, making it a novel perspective marker [53].

#### 3.1.8. Exosome

Exosomes were isolated from human urine, mononuclear THP-1 cell culture medium, and urothelial cell culture medium using ultracentrifugation and affinity chromatography [56]. The Akt and CD9 protein expression in the exosome was found to be surprisingly increased, reflecting that Akt and CD9 are expected to be helpful markers to distinguish UTI and ASB [56].

#### 3.1.9. Calprotectin

Calprotectin is a heterodimer composed of S100A8 and S100A9 proteins, whose concentrations in serum have been found to be significantly higher in cases than in controls, showing its substantial added value in distinguishing bacterial UTI in children under the age of 3 years [57]. It is worth noting that the latest research has demonstrated that urinary calprotectin is no less than dipstick pyuria in the detection of UTI [58].

### 3.2. Nucleic Acid-Associated Biomarkers

Molecular biology technology has made the detection of pathogenic microorganisms reach the level of DNA and RNA. In light of the timeliness and accuracy, cell-free DNA (cfDNA), transrenal DNA (Tr-DNA), and *16S ribosomal RNA* (rRNA) have been applied to the diagnosis of UTI.

#### 3.2.1. cfDNA and Tr-DNA

A mass of small fragments of cfDNA exists in urine and plasma [59]. Based on ‘omics principles’, it provides the opportunity for precise diagnosis, application to infection and cancer, as well as solid-organ transplantation [60]. Next-generation sequencing (NGS) has been performed in the study of cfDNA, and cfDNA was identified to be a versatile analyte for monitoring UTI [61].

Discovered as a category of extracellular urine DNA derived from dead cells, Tr-DNA is a type of cfDNA that crosses the renal barrier and appears in urine [62]. High concentrations of Tr-DNA were first found in urine specimens of UTI patients, and Tr-DNA quantification could produce a marked effect on clinical diagnosis through real-time quantitative PCR analysis [63].

#### 3.2.2. *16S rRNA*

*16S rRNA* gene, a molecular marker, only exists in the chromosomal genome of bacteria. Nine known hypervariable regions in *16S rRNA* amplicon sequencing were utilized to differentiate bacterial species via evolutionary polymorphisms [64], which provided higher sensitivity and specificity versus normal diagnostic methods. A study found that in the same collected urine samples, *16S rRNA* gene sequencing could detect 65.8% bacterial DNA, while standard urine culture showed a 90% false negative rate [65]. Another study showed that *16S rRNA* amplicon sequencing enabled the evaluation of the diversity of female urine flora, and the species resolution of pathogenic bacteria could be improved by detecting the urine of healthy women [66]. In addition, *16S rRNA* amplicon sequencing may assist in differential diagnosis when urine culture results are equivocal in children [67], and it is capable of helping identify major urinary microbiota genera related to UTI in vesicoureteral reflux [68]. Nevertheless, it was not generally efficient for *16S rRNA* amplicon sequencing among all taxa for its limitation in only capturing the primer-specified amplicon.

### 3.3. Glycosylated Markers

Compared with others, less is known regarding the glycosylated biomarkers in UTI. The studies mostly focus on sugar, such as sucrose, lactose, mannose, etc., and glycosyl compounds like glycosylated hemoglobin at present.

#### 3.3.1. Sucrose

Sucrose is the metabolite substantially elevated in the urine of *Chlamydia trachomatis*-positive (*CT*-positive) patients. The presence of urinary sucrose can not only increase the stability of chlamydia protein but also facilitate CT infection and enhance bacterial viability [69].

#### 3.3.2. Lactose and Other Metabolites

Lactose is known as a sign of bacterial metabolism. Unlike other Gram-negative bacteria causing UTI that produce succinate, acetate, and ethanol only and not lactate, *Escherichia coli* (*E. coli*) has a specific ability to metabolize lactose into lactate [70]. Gupta et al. developed a detection method based on nuclear magnetic resonance. In their procedure, the infected urine samples were incubated with the substrate for 6 h, and a decreased spectral resonance intensity of lactose and an increased spectral resonance intensity of lactate could be measured. The conversion of lactose to lactate in urine was utilized to identify and quantify the *E. coli* colonies in the urine of UTI patients [70]. In addition to lactate, other metabolites can be used to identify UTI pathogens by ^1^H-nuclear magnetic resonance spectroscopy (^1^H NMR spectroscopy). *Klebsiella pneumoniae* (*K. pneumoniae*) has the special property of metabolizing glycerol into 1,3-propanediol (1,3-PD), acetate, ethanol, and succinate, and there is a good correlation between the quantity of 1,3-PD produced and the viable bacterial count. Other common bacteria causing UTI are not able to metabolize glycerol under similar conditions, except for *Citrobacter frundii* (*C. frundii*). Although *C. frundii* also gives the same magnetic resonance results, *C. frundii* is easily distinguished as motile bacteria through direct urine microscopy examination, and it is not a common nosocomial source of UTI. Combining ^1^H NMR spectroscopy with direct urine microscopy examination to screen for *K. pneumoniae* and *C. frundii* in UTI had a sensitivity of 90% and specificity of 100% compared with the conventional quantitative culture method [71]. Similarly, *Pseudomonas aeruginosa* (*P. aeruginosa*) and *Proteus mirabilis* (*Pr. Mirabilis*) specifically metabolize nicotinic acid (NA) and methionine to 6-OHNA and 4-methylthio-2-oxobutyric acid (MOBA), respectively. The spectral resonance intensities of 6-OHNA and MOBA have been shown to correspond to the bacterial counts of *P. aeruginosa* and *Pr. Mirabilis* [70]. Using ^1^H NMR spectroscopy to assist in the identification and quantification of common UTI pathogens contributes to differentiating infected, contaminated, and sterile specimens and helping to address the shortcomings of urine culture in diagnosing UTI.

#### 3.3.3. HbA1c and O-Glycopeptide

In addition to the above sugars, the studies of glycosyl compounds as biomarkers in UTI also have broad prospects. Diabetic patients are apt to develop UTI more than non-diabetic individuals with more severe symptoms and consequences, which have four times the risk of complicating bacteremia than non-diabetic UTI patients [72]. Aswani et al. found elevated levels of glycosylated hemoglobin (HbA1c) in the blood predispose diabetics to UTI [72]. Blood HbA1c may assist in diagnosing complicated UTI caused by diabetic nephropathy. HbA1c was also proven to have the potential to diagnose the stage of chronic kidney disease [73]. Nevertheless, the role of elevated HbA1c concentrations in predicting UTI in diabetic patients may be related to their poor blood glucose control, and more studies are needed to prove its diagnostic value.

Another study collected samples from 39 patients with febrile UTI infected with *E. coli* and discovered a urinary O-glycopeptide for all the UTI patients, indicating its usefulness for diagnosing febrile UTI. To our knowledge, it is the first time for the demonstration of O-glycosylation of human fibrinogen α1-chain [74].

### 3.4. Metabolomic and Lipidomic Markers

With the global development of metabolomics and lipidomics, an increasing number of metabolites and lipidomic markers have been identified and applied to the diagnosis of UTI, which mainly include lactic acid, pyruvate, citrate, 3-hydroxybutyrate, leukocyte esterase (LE), nitrite (NIT), trimethylamine (TMA), acetate, creatinine, the siderocalin (SCN), siderophore, steroids, and phospholipids.

#### 3.4.1. Lactic Acid (Lactate) and Other Acids

Lactic acid, or lactate, is an outgrowth produced by highly proliferating cells in the glycolytic pathway, and it is also identified as the specific metabolite for *E. coli*. The motility of CD8^+^ and CD4^+^ T cells is constrained by extracellular lactic acid and sodium lactate, respectively. A further study confirmed that sodium lactate-mediated inhibition of CD4^+^ T cell motility is caused by interference with activated glycolysis, whereas the effect of lactic acid on CD8^+^ T cell motility has nothing to do with glycolysis control but leads to the loss of cytolytic function, which are the remarkable characteristics in chronic inflammatory infiltration of T cells [75]. A study showed active UTI patients had considerably higher levels of lactic acid in their urine [76]. Moreover, an obvious increase in the concentrations of lactate in urine samples of *CT*-positive subjects was detected by Foschi et al., resulting from *CT*-induced immunopathology [69].

In addition to lactate, pyruvate, the conjugate base of pyruvic acid, was also proved to increase in the urine of *CT*-positive subjects [69]. Citrate, among the intermediates in the TCA cycle, is generally considered to be an ‘uncomplicated’ metabolite but has an intricate network of functions. Urinary citrate excretion has been a measure of kidney function for many years, and the decrease in urine citrate of UTI patients on day t = 0 is a warning sign of renal metabolic pathological changes [77].

3-hydroxybutyrate has been regarded as a potential marker for its diagnostic value in assisting with UTI management. In UTI mouse models, the levels of 3-hydroxybutyrate decreased in plasma, with accuracy at 91%, sensitivity at 92%, and specificity at 91%, indicating 3-hydroxybutyrate was run out in blood, and it is closely relevant to UTI not only in the infection stage but also in the post-treatment stage [78].

#### 3.4.2. LE and NIT

LE is often used in urine tests to detect infection-related abnormalities. As other pathogen isolates cannot produce NIT from nitrate, NIT is regarded as being produced by *Enterobacteriaceae* [79]. Accordingly, whether the urine is bacteria-free should not be judged only by relying on a negative NIT test. Schwiemann et al. reported that the urine LE test (72%–94%) was measured to hold higher sensitivity than that of the urine NIT test (36%–57%), while the specificity of the LE test was lower among most adults [80]. The urine NIT test was demonstrated to have a positive predictive value of about 96% according to reports assisting in the diagnosis of gram-negative bacterial infection [81]. Besides, a study for male patients with clinical symptoms showed that a positive urine NIT test can be indicative of UTI [82]. Notably, the combination of NIT and LE tests in the urine brings about better performance than a single, providing an extraordinary screen for UTI [83].

#### 3.4.3. TMA

TMA is regarded as an ideal biomarker both for UTI and bacterial metabolic activity based on related research [84]. Bezabeh et al. proved that nine patients with UTI had elevated levels of TMA oxide. TMA can not only be used as a biomarker of UTI solo but also often combined with other metabolites. For example, urine TMA/creatinine levels have been identified as a specific marker of *E. coli*-associated UTI [85].

#### 3.4.4. Acetate (Acetic Acid) and Creatinine

Acetate has been thought to be an effective biomarker for all types of bacterial UTIs [86]. The 1H-NMR spectra were carried out by Lussu et al. on urine samples from different groups, and the results support the possibility of using acetate concentrations as a rapid test for diagnosis of *E. coli*-associated UTI [84]. However, absolute acetate concentrations in urine do not explain anything because of the different urine volumes of individuals. Creatinine is usually used to normalize the concentrations of compounds in urine, and the levels of these compounds were measured as a ratio to creatinine. According to Lam et al., while it was lower in the healthy group, the urine acetate/creatinine ratio reached 0.210 mg/1 mg in UTI patients. Regarded as a biomarker for bacterial UTI with excellent diagnostic value, the urinary acetic acid/creatinine levels had, overall accuracy, negative predictive value, positive predictive value, specificity, and sensitivity all exceeded 90%, outperforming microscopy examination or dipstick urinalysis [87].

#### 3.4.5. SCN and Siderophore

The antibacterial protein SCN, released by human urinary tract cells, is capable of interfering with the acquisition of iron in *E. coli* by chelating iron complexes with enterobactin. Additionally, SCN was detectable in all cystitis urine samples and correlated positively with enterobactin. Often expressing chemically distinctive siderophores, siderophores are widely present among the important bacteria. Using a metabolomics approach, Ohlemacher et al. ascertained *E. coli* secretes a second metallophore called escherichelin. After that, escherichelin was also detected in the urine of clinical *E. coli* UTI patients [88]. Furthermore, the regulation of siderophore is responsible for the distinct metabolome differences between the UPEC and non-UPEC strains. The result showed that siderophore biosynthesis harmonizes the differential metabolome, providing new targets for diagnosis, therapy, and drug discovery related to UTI [89].

#### 3.4.6. Steroids

In recent years, the research on steroids in UTI has been carried out gradually. Samples were screened and categorized by Adebayo et al., and they principally found levels of host sex steroids in cases with urogenital schistosomiasis alone and cases with urogenital schistosomiasis-related bladder pathologies were shown to be low, since some research had shown that the growth of flatworms may extensively rely on the host sex steroids [90,91]. Catechol estrogen was also proved to be related to UTI caused by parasites, possessing a diagnosis value for distinguishing infection-only from advanced cases [90]. Another study identified compounds associated with *E. coli* UTI, showing higher urine levels of two metabolites that are involved in steroid biosynthesis and lower levels of three glucuronic steroid metabolites in the UTI group. Although this pattern represents the host response to elevated steroid hormone levels, it probably reflects the bacterial metabolism of pathogens, as *E. coli* cannot synthesize steroids, but they are known to have a glucuronidase that can degrade glucuronide-acidified compounds, indicating that there may be endocrine interactions between hosts and pathogens [92].

As a type of steroid, the diagnostic value of vitamin D (VD) in UTI has attracted the attention of scholars. A study has lately found that VD mediates the link between innate and adaptive immune responses to UPEC infection by inducing homologous regulation of Th1/Th17 polarization of cytokine responses and antibody production, as well as maintaining alternative complement pathways [93]. Using serum 25-hydroxyvitamin D (25OHD) to explore the relationship between UTI in adults and the levels of VD, Liu et al. detected that the concentrations of serum 25OHD in female UTI patients were significantly lower than the control group, and there was no correlation between the concentrations of serum 25OHD and UTI among male patients [94]. In particular, VD deficiency is recognized as a risk factor for UTI in females at reproductive age [95]. Furthermore, similar conclusions were found in the evaluation of risk factors for UTI among children. The results demonstrated that the serum VD concentrations in children with UTI were markedly lower than those in the children without UTI, showing a negative association existed between serum concentrations and UTI risk in children [96].

#### 3.4.7. Phospholipids

Compared to other phospholipids, glycerophospholipids and sphingolipids are more often used in the diagnosis of UTI. Alternative markers to distinguish between sepsis and systemic inflammatory response syndrome were identified by a study, analyzing a total of 406 patients, 112 of whom had UTI. They determined that compared to patients with severe intra-abdominal infection, the severe UTI group had increased levels of three glycerophospholipids in serum, and higher concentrations of two biogenic amines, six glycerophospholipids, and two sphingolipids and lower concentrations of two glycerophospholipids in serum were shown in the severe UTI group in comparison with the severe bloodstream infection group, indicating glycerophospholipids and sphingolipids may assist in the diagnosis of sepsis caused by severe UTI [97].

## 4. Controversial Biomarkers

Varying in the research methods, there are some biomarkers verified to be controversial in UTI diagnosis and require more high-standard prospective studies.

### 4.1. TREM1

Belonging to the immunoglobulin superfamily, the triggering receptor expressed on myeloid cell 1 (TREM-1) is expressed in macrophages and neutrophils [98] and exists as a soluble form (STREM-1) in body fluids. Though STREM-1 shows diagnostic value in many bacterial infections, an early study demonstrated the impracticality of urine STREM-1 in UTI due to its low concentration and sensitivity [99]. Interestingly, Sierra-Diaz et al. found higher expression of TREM-1, not the soluble form, by flow cytometry in pediatric patients with UTI. This study concluded that TREM-1 in urinary cells may be a useful marker of UTI diagnosis in children [100]. On the other hand, serum STREM1 shows better diagnostic value. It was demonstrated that serum STREM-1 achieved a sensitivity of 97.3% and specificity of 84% for diagnosing APN with a cut-off of 127.37 pg/mL [101]. But in another study, researchers found that serum STREM-1 helped to diagnose normal cases with false positive urine culture or suspected upper UTI cases with false-negative urine culture, while its value in discriminating the upper from lower UTI was limited [102].

### 4.2. D-Dimer

D-dimer is a symbol of the coagulation system activation and serves as a marker in numerous systematic diseases. The diagnostic performance of D-dimer in UTI is poorly studied to date. Lee et al. discovered that serum D-dimer was highly upregulated in acute inflammation and might be a potential marker for APN in infants [103]. A recent study conducted by Esteghamati and her colleagues revealed similar results, in which the serum D-dimer positive was observed in 81.4% of children aged 1 month to 14 years; meanwhile, they declared that D-dimer showed better than PCT in detecting upper UTI and the renal parenchymal involvement [104]. However, Liu et al. reported a case of scrub typhus presenting as a UTI with upregulated levels of D-dimer [105]. In general, D-dimer is more suggestive of renal parenchymal involvement and other complications in UTI.

### 4.3. CCL3

The chemokine CCL3 has been investigated as a potential UTI marker in both mice and human models, yet the available data yield conflicting results. Selected studies have shown elevated CCL3 concentrations in UTI [106], whereas others failed to detect distinguishable differences in urinary CCL3 levels [107]. Of note, a recent study reported great sensitivity and specificity of urinary CCL3 for differentiating febrile children with and without UTI [108].

### 4.4. MR-proADM

Mid-regional proadrenomedullin (MR-proADM) is a well-explored parameter in sepsis, and studies have investigated its utility in UTI [109]. Stalenhoef et al. verified that plasma MR-proADM had a better ability to precisely predict the progression of severe febrile UTI compared with both PCT and CRP, especially, which is conducive to guiding UTI emergency triage [110]. While one later study revealed contradictory results, suggesting neither plasma nor urinary MR-proADM showed prognostic value in UTI, the median of plasmatic MR-proADM was useful for renal scar detection [111].

### 4.5. Human Neutrophil Peptides

Human Neutrophil Peptides 1, 2, and 3 (HNP1-3) are members ofα-defensin family and perform effectively as powerful antimicrobial peptides (AMPs) in protecting against UTI [112]. A previous study noted that urine HNP1-3 and HD-5 (β-defensin family) were promising AMP biomarkers with high sensitivity for predicting positive urine culture in UTI patients [113]. Nevertheless, Shaikh et al. reported converse results, as they found no difference in urinary HNP1 levels (nor its gene DEFA1) in healthy and UTI groups [106].

### 4.6. Others

YKL-40 refers to cartilage glycoprotein 40 and belongs to the family of mammalian chitinase-like proteins. Early studies revealed the potential of plasma YKL-40 in nephropathy diagnosis [114,115], but its role in UTI is still ambiguous with rare evidence [116].

## 5. Conclusions and Future Perspectives

Given the limitations of traditional UTI diagnostic methods in certain clinical contexts, there is increasing interest in identifying biomarkers that offer rapid, sensitive, and accurate diagnostic capabilities. According to our study, NGAL is an effective biomarker for UTI and has been widely used in recent years. IL-1β, IL-8, and HBP may facilitate the differentiation of upper and lower UTI, particularly in the case of APNs. MMP-9 and LF may aid in the diagnosis of ASB, but further studies and investment are required to fully realize their potential. Besides, promising recent developments in the exploration of nucleic acid-associated biomarkers such as *16S rRNA* offer the potential for identifying pathogenic microorganisms, with exciting prospects given the growing interest in urinary microbiome and metagenomic sequencing studies. Additionally, the potential role of metabolic and lipidomic markers in UTI diagnostics represents a compelling area for further exploration.

To sum up, numerous biomarkers have been studied, but, in general, opportunities co-exist with challenges. Variation in the sensitivity and specificity of different biomarkers derived from factors such as age, gender, and underlying health conditions further complicates the issue. As such, well-designed clinical studies and new diagnostic tests are necessary in the future. While looking for reliable markers, microbiological diagnosis continues to play a basic role in UTI monitoring and diagnosis. We believe the effective combination of the two will bring dawn to the diagnosis and therapy of UTI.

## Figures and Tables

**Table 1:** Biomarkers used clinically for UTI.

Biomarkers	Sample	Sensitivity	Specificity	ASB Significance	Clinical Implication	Reference
IL-1β	Serum	97%	59%	-	a marker for detecting upper UTI and pyelonephritis in children	[11]
	Urine	88%	79%	-	a marker for early detecting acute pyelonephritis in febrile children	[12]
IL-6	Serum	60%	55%	-	possesses a diagnostic value to differentiate UTI in both children and adults	[13,14]
	Urine	57%	80%	+	assists the detection between ASB and symptomatic UTI in the elderly	[15]
IL-8	Urine	93%	60%	-	holds the diagnostic value for UTI in children	[14,16]
PCT	Serum	81%	76%	-	a suitable biomarker for differential diagnosis in children has a role in predicting pyelonephritis	[17,18,19]
CRP	Serum	93%	37%	-	detection of lower UTI and upper UTI in children use CRP levels to exclude pyelonephritis	[17,18]
NGAL	Urine	90%	94%	-	a predictive marker for recurrent UTI a marker for the diagnosis of UTI in adults and infants	[20,21,22]

## Data Availability

Not applicable.

## Abbreviations

UTI, Urinary tract infection; PCR, polymerase chain reaction; UPEC, uropathogenic *Escherichia coli*; PCT, Procalcitonin; CRP, C-reactive protein; NPV, negative predictive value; APN, acute pyelonephritis; ASB, asymptomatic bacteriuria; NGAL, Neutrophil gelatinase-associated lipocalin; HBP, Heparin-binding protein; MMP-9, Matrix metalloproteinases-9; TIMP1, tissue inhibitor of metalloproteinase 1; VUR, vesicoureteral reflux; LF, Lactoferrin; HSP70, Heat shock protein-70; BMP-2, Bone morphogenic protein-2; CysC, Cystatin C; THP, Tamm-Horsfall protein; MPO, Myeloperoxidase; NETs, neutrophil extracellular traps; XO, xanthine oxidase; cfDNA, cell-free DNA; Tr-DNA, transrenal DNA; rRNA, ribosomal RNA; NGS, next-generation sequencing; *CT*-positive, *Chlamydia trachomatis* positive; *E. coli*, *Escherichia coli*; ^1^H NMR spectroscopy, ^1^H-nuclear magnetic resonance spectroscopy; *K. pneumoniae*, *Klebsiella pneumoniae*; 1,3-PD, 1,3-propanediol; *C. frundii*, *Citrobacter frundii*; *P. aeruginosa*, *Pseudomonas aeruginosa*; *Pr. Mirabilis*, *Proteus mirabilis*; NA, nicotinic acid; MOBA, 4-methylthio-2-oxobutyric acid; HbA1c, glycosylated hemoglobin; LE, leukocyte esterase; NIT, nitrite; TMA, trimethylamine; SCN, trimethylamine; VD, vitamin D; 25OHD, 25-hydroxyvitamin D; TREM-1, triggering receptor expressed on myeloid cell 1; MR-proADM, Mid-regional proadrenomedullin; HNP1-3, Human Neutrophil Peptides 1, 2, and 3; AMPs, antimicrobial peptides.
